# 4-[3-(1-Naphthyl­oxymeth­yl)-7*H*-1,2,4-triazolo[3,4-*b*][1,3,4]thia­diazin-6-yl]-3-*p*-tolyl­sydnone

**DOI:** 10.1107/S1600536810017812

**Published:** 2010-05-22

**Authors:** Jia Hao Goh, Hoong-Kun Fun, Nithinchandra, B. Kalluraya

**Affiliations:** a X-ray Crystallography Unit, School of Physics, Universiti Sains Malaysia, 11800 USM, Penang, Malaysia; b Department of Studies in Chemistry, Mangalore University, Mangalagangotri, Mangalore 574 199, India

## Abstract

In the title sydnone compound, C_24_H_18_N_6_O_3_S {systematic name: 4-[3-(1-naphthyl­oxymeth­yl)-7*H*-1,2,4-triazolo[3,4-*b*][1,3,4]thia­diazin-6-yl]-3-*p*-tolyl-4,5-dihydro-1,2,3-oxadiazol-3-ium-5-olate} an intra­molecular C—H⋯O hydrogen bond generates an *S*(6) ring motif. The 3,6-dihydro-1,3,4-thia­diazine ring adopts a twist-boat conformation. The essentially planar 1,2,3-oxadiazole and 1,2,4-triazole rings [maximum deviations of 0.006 (1) and 0.008 (1) Å, respectively] are inclined to one another at inter­planar angle of 44.11 (4)°. The naphthalene unit forms an inter­planar angle of 66.40 (4)° with the 1,2,4-triazole ring. In the crystal packing, pairs of inter­molecular C—H⋯O hydrogen bonds link adjacent mol­ecules into dimers incorporating *R*_2_^2^(12) ring motifs. Further stabilization is provided by weak C—H⋯π inter­actions.

## Related literature

For general background to and applications of the title sydnone compound, see: Baker *et al.* (1949 ▶); Hedge *et al.* (2008 ▶); Rai *et al.* (2008 ▶). For graph-set descriptions of hydrogen-bond ring motifs, see: Bernstein *et al.* (1995 ▶). For related structures, see: Baker & Ollis (1957 ▶); Goh *et al.* (2010**a* ▶,*b* ▶,c* ▶). For the stability of the temperature controller used for the data collection, see: Cosier & Glazer (1986 ▶).
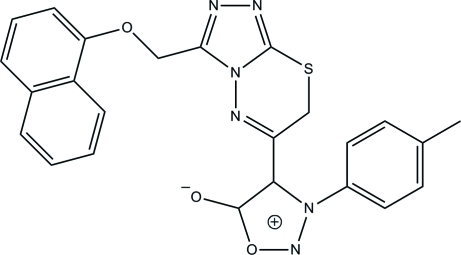


## Experimental

### 

#### Crystal data

C_24_H_18_N_6_O_3_S
*M* *_r_* = 470.50
Monoclinic, 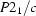

*a* = 21.6096 (8) Å
*b* = 8.3622 (3) Å
*c* = 11.9272 (4) Å
β = 94.694 (1)°
*V* = 2148.06 (13) Å^3^
*Z* = 4
Mo *K*α radiation
μ = 0.19 mm^−1^
*T* = 100 K
0.82 × 0.28 × 0.22 mm

#### Data collection

Bruker APEXII DUO CCD area-detector diffractometer
Absorption correction: multi-scan (*SADABS*; Bruker, 2009 ▶) *T*_min_ = 0.857, *T*_max_ = 0.959
30407 measured reflections
9432 independent reflections
8048 reflections with *I* > 2σ(*I*)
*R*_int_ = 0.023

#### Refinement

*R*[*F* ^2^ > 2σ(*F* ^2^)] = 0.036
*wR*(*F* ^2^) = 0.110
*S* = 1.08
9432 reflections
379 parameters
All H-atom parameters refined
Δρ_max_ = 0.58 e Å^−3^
Δρ_min_ = −0.31 e Å^−3^

### 

Data collection: *APEX2* (Bruker, 2009 ▶); cell refinement: *SAINT* (Bruker, 2009 ▶); data reduction: *SAINT*; program(s) used to solve structure: *SHELXTL* (Sheldrick, 2008 ▶); program(s) used to refine structure: *SHELXTL*; molecular graphics: *SHELXTL*; software used to prepare material for publication: *SHELXTL* and *PLATON* (Spek, 2009 ▶).

## Supplementary Material

Crystal structure: contains datablocks global, I. DOI: 10.1107/S1600536810017812/lh5045sup1.cif

Structure factors: contains datablocks I. DOI: 10.1107/S1600536810017812/lh5045Isup2.hkl

Additional supplementary materials:  crystallographic information; 3D view; checkCIF report

## Figures and Tables

**Table 1:** Hydrogen-bond geometry (Å, °) *Cg*1 and *Cg*2 are the centroids of the C18–C23 and C1/C6–C10 benzene rings, respectively.

*D*—H⋯*A*	*D*—H	H⋯*A*	*D*⋯*A*	*D*—H⋯*A*
C14—H14*B*⋯O3	0.973 (13)	2.401 (14)	3.0928 (10)	127.7 (10)
C14—H14*B*⋯O3^i^	0.973 (13)	2.396 (13)	3.1612 (10)	135.2 (11)
C8—H8*A*⋯*Cg*1^ii^	0.980 (17)	2.603 (17)	3.3928 (11)	138.0 (13)
C24—H24*C*⋯*Cg*2	0.993 (19)	2.95 (2)	3.7066 (14)	134.3 (17)

Symmetry codes: (i) 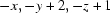; (ii) 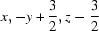.

**Fig. 1.**
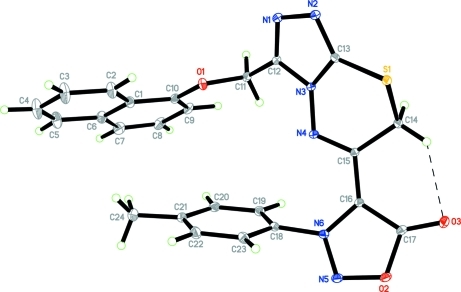
The molecular structure of the title compound, showing 30% probability displacement ellipsoids for non-H atoms and the atom-numbering scheme. An intramolecular hydrogen bond is shown as dashed line.

**Fig. 2.**
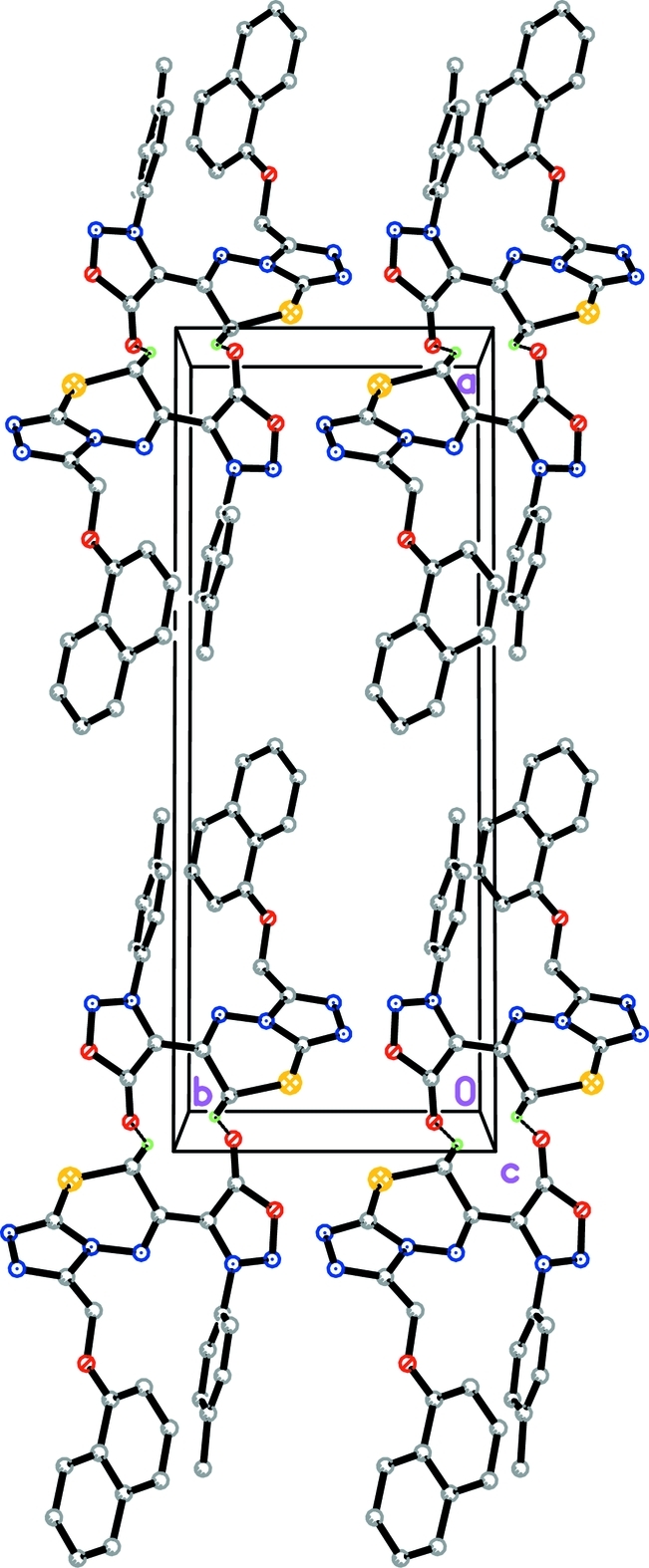
The crystal structure of the title compound, viewed along the c axis, showing adjacent molecules being linked into dimers. Hydrogen atoms not involved in intermolecular interactions (dashed lines) have been omitted for clarity.

## supplementary crystallographic information

### Comment

Sydnones constitute a well-defined class of mesoionic compounds consisting of 1,2,3-oxadiazole ring system. The introduction of the concept of mesoionic structure for certain heterocyclic compounds in the year 1949 has proved to be a fruitful development in heterocyclic chemistry (Baker *et al.*, 1949). The study of sydnones still remains a field of interest because of their electronic structures and also because of the various types of biological activities displayed by some of them. Interest in sydnone derivatives has also been encouraged by the discovery that they exhibit various pharmacological activities (Hedge *et al.*, 2008; Rai *et al.*, 2008).

A series of triazolothiadiazines were synthesized by the condensation of 4-bromoacetyl-3-aryl sydnones with 3-aryloxymethyl-4-amino-5-mercapto-1,2,4-triazoles. 4-Bromoacetyl-3-aryl sydnones were in turn obtained by the photochemical bromination of 4-acetyl-3-aryl sydnones. 3-Aryloxymethyl-4-amino-5-mercapto-1,2,4-triazoles were prepared, starting from the potassium salt of aryloxyacetyl hydrazines with carbon disulphide in alcoholic KOH. The hydrazides were in turn prepared from the corresponding esters. These aryloxymethyl esters were prepared by the reaction of appropriately substituted phenol with ethylchloroacetate in presence of anhydrous potassium carbonate in dry acetone medium.

In the title sydnone compound, an intramolecular C14—H14B···O3 hydrogen bond (Table 1) generates a six-membered ring, producing an *S*(6) hydrogen bond ring motif (Fig. 1, Bernstein *et al.*, 1995). The 3,6-dihydro-1,3,4-thiadiazine ring adopts a twist-boat conformation, with puckering parameters of Q = 0.6251 (7) Å, θ = 112.76 (6)° and φ = 142.75 (7)° (Cremer & Pople, 1975). The 1,2,3-oxadiazole (C16/C17/O2/N5/N6) and 1,2,4-triazole (C12/N1/N2/C13/N3) rings are essentially planar, with maximum deviations of 0.006 (1) and -0.008 (1) Å, respectively, at atoms C16 and N1. The interplanar angle between these two rings is 44.11 (4)°. The C18–C23 benzene ring and C1–C10 naphthalene ring system are making interplanar angles of 69.72 (4) and 66.40 (4)°, with the 1,2,3-oxadiazole and 1,2,4-triazole rings, respectively. As reported previously (Goh *et al.*, 2010*a,b*), the exocyclic C17–O3 bond length [1.2126 (9) Å] of the sydnone unit is inconsistent with the formulation of Baker & Ollis (1957), which reported the delocalization of a positive charge in the 1,2,3-oxadiazole ring, and a negative charge in the exocyclic oxygen. The bond lengths and angles are within normal ranges and comparable to those reported in closely related sydnone (Goh *et al.*, 2010*a,b*) and 1,2,4-triazole (Goh *et al.*, 2010*c*) structures.

In the crystal packing, pairs of intermolecular C14—H14B···O3[-x, -y+2, -z+1] hydrogen bonds (Table 1) link adjacent molecules into dimers incorporating *R*^2^_2_(12) ring motifs (Fig. 2, Bernstein *et al.*, 1995). Further stabilization is provided by weak C8—H8A···*Cg*1 [x, -y+3/2, z-3/2] and C24—H24C···*Cg*2 interactions (Table 1) involving the C18-C23 (*Cg*1) and C1/C6-C10 (*Cg*2) benzene rings.

### Experimental

An equimolar mixture of 3-naphthyloxymethyl-4-amino-5-mercapto-1,2,4-triazoles (0.01 mol) and 4-bromoacetyl-3-tolylsydnones (0.01 mol) in absolute ethanol was heated under reflux for 10-12 h. The solution was concentrated, cooled to room temperature and neutralized with 10 % sodium bicarbonate solution. Solid product formed was collected by filtration and recrystallized from ethanol. Single crystals for X-ray analysis were obtained from a 1:2 mixture of DMF and ethanol by slow evaporation.

### Refinement

All hydrogen atoms were located from difference Fourier map [range of C—H = 0.945 (17)–1.028 (16) Å] and allowed to refine freely.

### Crystal data

**Table:**

C_24_H_18_N_6_O_3_S	*F*(000) = 976
*M**_r_* = 470.50	*D*_x_ = 1.455 Mg m^−^^3^
Monoclinic, *P*2_1_/*c*	Mo *K*α radiation, λ = 0.71073 Å
Hall symbol: -P 2ybc	Cell parameters from 9918 reflections
*a* = 21.6096 (8) Å	θ = 2.6–35.1°
*b* = 8.3622 (3) Å	µ = 0.19 mm^−^^1^
*c* = 11.9272 (4) Å	*T* = 100 K
β = 94.694 (1)°	Block, colourless
*V* = 2148.06 (13) Å^3^	0.82 × 0.28 × 0.22 mm
*Z* = 4	

### Data collection

**Table:**

Bruker APEXII DUO CCD area-detector diffractometer	9432 independent reflections
Radiation source: fine-focus sealed tube	8048 reflections with *I* > 2σ(*I*)
graphite	*R*_int_ = 0.023
φ and ω scans	θ_max_ = 35.1°, θ_min_ = 1.9°
Absorption correction: multi-scan (*SADABS*; Bruker, 2009)	*h* = −34→34
*T*_min_ = 0.857, *T*_max_ = 0.959	*k* = −13→11
30407 measured reflections	*l* = −19→17

### Refinement

**Table:**

Refinement on *F*^2^	Primary atom site location: structure-invariant direct methods
Least-squares matrix: full	Secondary atom site location: difference Fourier map
*R*[*F*^2^ > 2σ(*F*^2^)] = 0.036	Hydrogen site location: inferred from neighbouring sites
*wR*(*F*^2^) = 0.110	All H-atom parameters refined
*S* = 1.08	*w* = 1/[σ^2^(*F*_o_^2^) + (0.0621*P*)^2^ + 0.4207*P*] where *P* = (*F*_o_^2^ + 2*F*_c_^2^)/3
9432 reflections	(Δ/σ)_max_ = 0.001
379 parameters	Δρ_max_ = 0.58 e Å^−^^3^
0 restraints	Δρ_min_ = −0.31 e Å^−^^3^

### Special details

**Table:**

Experimental. The crystal was placed in the cold stream of an Oxford Cryosystems Cobra open-flow nitrogen cryostat (Cosier & Glazer, 1986) operating at 100.0 (1)K.
Geometry. All esds (except the esd in the dihedral angle between two l.s. planes) are estimated using the full covariance matrix. The cell esds are taken into account individually in the estimation of esds in distances, angles and torsion angles; correlations between esds in cell parameters are only used when they are defined by crystal symmetry. An approximate (isotropic) treatment of cell esds is used for estimating esds involving l.s. planes.
Refinement. Refinement of F^2^ against ALL reflections. The weighted R-factor wR and goodness of fit S are based on F^2^, conventional R-factors R are based on F, with F set to zero for negative F^2^. The threshold expression of F^2^ > 2sigma(F^2^) is used only for calculating R-factors(gt) etc. and is not relevant to the choice of reflections for refinement. R-factors based on F^2^ are statistically about twice as large as those based on F, and R- factors based on ALL data will be even larger.

### Fractional atomic coordinates and isotropic or equivalent isotropic displacement parameters (Å^2^)

**Table:**

	*x*	*y*	*z*	*U*_iso_*/*U*_eq_	
S1	0.054237 (8)	0.64796 (2)	0.368050 (17)	0.01458 (5)	
O1	0.25803 (3)	0.72732 (8)	0.06654 (5)	0.01628 (11)	
O2	0.09850 (3)	1.30606 (7)	0.46932 (5)	0.01662 (11)	
O3	0.00745 (3)	1.17152 (8)	0.43365 (5)	0.01799 (11)	
N1	0.14829 (3)	0.50393 (9)	0.12173 (6)	0.01643 (12)	
N2	0.11061 (3)	0.46927 (8)	0.20910 (6)	0.01603 (12)	
N3	0.12737 (3)	0.72984 (8)	0.20468 (6)	0.01263 (11)	
N4	0.13693 (3)	0.88576 (8)	0.24115 (6)	0.01300 (11)	
N5	0.15806 (3)	1.29814 (8)	0.43898 (6)	0.01614 (12)	
N6	0.15941 (3)	1.17335 (8)	0.37354 (5)	0.01269 (11)	
C1	0.35987 (4)	0.77223 (11)	0.01602 (7)	0.01752 (14)	
C2	0.38308 (4)	0.65856 (13)	0.09650 (8)	0.02498 (18)	
C3	0.44556 (5)	0.62503 (19)	0.10958 (11)	0.0366 (3)	
C4	0.48733 (5)	0.7049 (2)	0.04353 (11)	0.0389 (3)	
C5	0.46585 (5)	0.81610 (17)	−0.03400 (10)	0.0320 (2)	
C6	0.40179 (4)	0.85376 (12)	−0.05035 (8)	0.02161 (16)	
C7	0.37898 (5)	0.97005 (13)	−0.12929 (8)	0.02567 (18)	
C8	0.31662 (5)	0.99837 (12)	−0.14608 (8)	0.02419 (17)	
C9	0.27387 (4)	0.91590 (10)	−0.08351 (7)	0.01914 (14)	
C10	0.29522 (4)	0.80784 (10)	−0.00217 (7)	0.01503 (13)	
C11	0.19294 (3)	0.75150 (10)	0.04109 (7)	0.01550 (13)	
C12	0.15819 (3)	0.65872 (9)	0.12197 (7)	0.01378 (12)	
C13	0.09846 (3)	0.60679 (9)	0.25608 (7)	0.01354 (12)	
C14	0.03560 (3)	0.85027 (9)	0.31981 (7)	0.01418 (12)	
C15	0.09438 (3)	0.94058 (9)	0.30182 (6)	0.01176 (11)	
C16	0.10454 (3)	1.09342 (9)	0.35705 (6)	0.01216 (11)	
C17	0.06224 (4)	1.18116 (9)	0.41931 (6)	0.01406 (12)	
C18	0.21821 (3)	1.14204 (9)	0.32812 (6)	0.01316 (12)	
C19	0.22339 (4)	1.16937 (10)	0.21476 (7)	0.01628 (13)	
C20	0.28120 (4)	1.14737 (11)	0.17364 (7)	0.01943 (15)	
C21	0.33262 (4)	1.10009 (11)	0.24445 (8)	0.02073 (15)	
C22	0.32506 (4)	1.07116 (11)	0.35765 (8)	0.02126 (15)	
C23	0.26765 (4)	1.09216 (10)	0.40109 (7)	0.01746 (14)	
C24	0.39559 (5)	1.08553 (16)	0.19990 (11)	0.0326 (2)	
H2A	0.3546 (7)	0.602 (2)	0.1375 (14)	0.036 (4)*	
H3A	0.4629 (9)	0.548 (3)	0.1663 (17)	0.056 (5)*	
H4A	0.5305 (9)	0.677 (3)	0.0521 (16)	0.053 (5)*	
H5A	0.4945 (7)	0.876 (2)	−0.0788 (14)	0.033 (4)*	
H7A	0.4102 (7)	1.029 (2)	−0.1753 (13)	0.035 (4)*	
H8A	0.3011 (7)	1.076 (2)	−0.2028 (14)	0.038 (4)*	
H9A	0.2288 (6)	0.9413 (19)	−0.0985 (11)	0.024 (3)*	
H11A	0.1812 (6)	0.7163 (18)	−0.0368 (11)	0.020 (3)*	
H11B	0.1818 (6)	0.8606 (17)	0.0462 (12)	0.021 (3)*	
H14A	0.0118 (6)	0.8427 (17)	0.2483 (12)	0.020 (3)*	
H14B	0.0114 (6)	0.8965 (17)	0.3770 (11)	0.017 (3)*	
H19A	0.1867 (6)	1.1991 (18)	0.1683 (12)	0.022 (3)*	
H20A	0.2844 (7)	1.1636 (18)	0.0939 (13)	0.026 (3)*	
H22A	0.3595 (7)	1.039 (2)	0.4079 (12)	0.029 (4)*	
H23A	0.2619 (6)	1.0695 (19)	0.4794 (12)	0.026 (3)*	
H24A	0.4217 (10)	1.175 (3)	0.2286 (18)	0.059 (6)*	
H24B	0.4133 (8)	0.985 (3)	0.2221 (16)	0.049 (5)*	
H24C	0.3950 (8)	1.088 (3)	0.1166 (16)	0.048 (5)*	

### Atomic displacement parameters (Å^2^)

**Table:**

	*U*^11^	*U*^22^	*U*^33^	*U*^12^	*U*^13^	*U*^23^
S1	0.01306 (8)	0.01134 (8)	0.01984 (9)	−0.00084 (5)	0.00436 (6)	0.00005 (6)
O1	0.0125 (2)	0.0195 (3)	0.0170 (2)	0.00011 (19)	0.00242 (18)	0.0030 (2)
O2	0.0204 (2)	0.0124 (2)	0.0174 (3)	0.00013 (19)	0.00400 (19)	−0.00343 (19)
O3	0.0160 (2)	0.0173 (3)	0.0211 (3)	0.00308 (19)	0.0046 (2)	−0.0018 (2)
N1	0.0154 (3)	0.0131 (3)	0.0211 (3)	0.0009 (2)	0.0029 (2)	−0.0035 (2)
N2	0.0145 (2)	0.0116 (3)	0.0221 (3)	0.0004 (2)	0.0027 (2)	−0.0025 (2)
N3	0.0116 (2)	0.0099 (2)	0.0166 (3)	−0.00056 (18)	0.00252 (19)	−0.0028 (2)
N4	0.0138 (2)	0.0095 (2)	0.0160 (3)	−0.00074 (19)	0.0026 (2)	−0.0023 (2)
N5	0.0197 (3)	0.0124 (3)	0.0165 (3)	−0.0018 (2)	0.0026 (2)	−0.0030 (2)
N6	0.0151 (2)	0.0103 (2)	0.0127 (2)	−0.00127 (19)	0.00128 (19)	−0.00056 (19)
C1	0.0156 (3)	0.0205 (4)	0.0168 (3)	−0.0006 (3)	0.0035 (2)	0.0003 (3)
C2	0.0160 (3)	0.0350 (5)	0.0239 (4)	0.0015 (3)	0.0015 (3)	0.0093 (4)
C3	0.0175 (4)	0.0562 (8)	0.0361 (5)	0.0061 (4)	0.0015 (4)	0.0177 (5)
C4	0.0160 (4)	0.0603 (8)	0.0409 (6)	0.0026 (4)	0.0059 (4)	0.0126 (6)
C5	0.0191 (4)	0.0453 (6)	0.0330 (5)	−0.0026 (4)	0.0101 (3)	0.0052 (5)
C6	0.0192 (3)	0.0255 (4)	0.0211 (4)	−0.0014 (3)	0.0076 (3)	0.0003 (3)
C7	0.0299 (4)	0.0239 (4)	0.0250 (4)	−0.0017 (3)	0.0131 (3)	0.0031 (3)
C8	0.0317 (4)	0.0186 (4)	0.0238 (4)	0.0049 (3)	0.0116 (3)	0.0057 (3)
C9	0.0232 (3)	0.0163 (3)	0.0187 (3)	0.0054 (3)	0.0064 (3)	0.0023 (3)
C10	0.0163 (3)	0.0147 (3)	0.0145 (3)	0.0004 (2)	0.0043 (2)	−0.0007 (2)
C11	0.0135 (3)	0.0165 (3)	0.0167 (3)	0.0022 (2)	0.0025 (2)	−0.0007 (2)
C12	0.0127 (3)	0.0127 (3)	0.0161 (3)	0.0013 (2)	0.0021 (2)	−0.0032 (2)
C13	0.0108 (2)	0.0109 (3)	0.0189 (3)	−0.0006 (2)	0.0010 (2)	−0.0012 (2)
C14	0.0111 (3)	0.0116 (3)	0.0199 (3)	0.0005 (2)	0.0022 (2)	−0.0014 (2)
C15	0.0114 (2)	0.0099 (3)	0.0140 (3)	0.0004 (2)	0.0010 (2)	−0.0006 (2)
C16	0.0130 (3)	0.0099 (3)	0.0138 (3)	−0.0001 (2)	0.0023 (2)	−0.0011 (2)
C17	0.0171 (3)	0.0111 (3)	0.0142 (3)	0.0018 (2)	0.0023 (2)	−0.0007 (2)
C18	0.0131 (3)	0.0115 (3)	0.0149 (3)	−0.0018 (2)	0.0018 (2)	−0.0002 (2)
C19	0.0180 (3)	0.0167 (3)	0.0142 (3)	−0.0019 (2)	0.0018 (2)	−0.0010 (2)
C20	0.0211 (3)	0.0196 (4)	0.0182 (3)	−0.0039 (3)	0.0059 (3)	−0.0048 (3)
C21	0.0164 (3)	0.0175 (3)	0.0289 (4)	−0.0023 (3)	0.0054 (3)	−0.0079 (3)
C22	0.0153 (3)	0.0194 (4)	0.0286 (4)	−0.0002 (3)	−0.0009 (3)	0.0002 (3)
C23	0.0169 (3)	0.0162 (3)	0.0189 (3)	−0.0019 (2)	−0.0010 (2)	0.0029 (3)
C24	0.0192 (4)	0.0360 (6)	0.0440 (6)	−0.0028 (4)	0.0112 (4)	−0.0160 (5)

### Geometric parameters (Å, °)

**Table:**

S1—C13	1.7390 (8)	C7—C8	1.3667 (14)
S1—C14	1.8214 (8)	C7—H7A	1.028 (16)
O1—C10	1.3707 (10)	C8—C9	1.4138 (12)
O1—C11	1.4289 (9)	C8—H8A	0.980 (17)
O2—N5	1.3667 (9)	C9—C10	1.3775 (12)
O2—C17	1.4086 (10)	C9—H9A	0.998 (14)
O3—C17	1.2126 (9)	C11—C12	1.4882 (11)
N1—C12	1.3119 (10)	C11—H11A	0.988 (14)
N1—N2	1.4044 (10)	C11—H11B	0.946 (14)
N2—C13	1.3151 (10)	C14—C15	1.5080 (10)
N3—C12	1.3704 (10)	C14—H14A	0.962 (14)
N3—C13	1.3739 (10)	C14—H14B	0.974 (13)
N3—N4	1.3846 (9)	C15—C16	1.4464 (10)
N4—C15	1.2994 (9)	C16—C17	1.4272 (10)
N5—N6	1.3049 (9)	C18—C19	1.3849 (11)
N6—C16	1.3612 (9)	C18—C23	1.3860 (11)
N6—C18	1.4454 (10)	C19—C20	1.3910 (12)
C1—C2	1.4134 (13)	C19—H19A	0.962 (14)
C1—C6	1.4246 (12)	C20—C21	1.3967 (13)
C1—C10	1.4275 (11)	C20—H20A	0.969 (15)
C2—C3	1.3752 (13)	C21—C22	1.3943 (14)
C2—H2A	0.945 (17)	C21—C24	1.5058 (13)
C3—C4	1.4134 (16)	C22—C23	1.3941 (12)
C3—H3A	0.99 (2)	C22—H22A	0.954 (15)
C4—C5	1.3659 (18)	C23—H23A	0.971 (14)
C4—H4A	0.96 (2)	C24—H24A	0.98 (2)
C5—C6	1.4174 (14)	C24—H24B	0.96 (2)
C5—H5A	0.986 (16)	C24—H24C	0.993 (19)
C6—C7	1.4139 (14)		
			
C13—S1—C14	93.58 (4)	C12—C11—H11B	108.4 (8)
C10—O1—C11	114.75 (6)	H11A—C11—H11B	107.5 (12)
N5—O2—C17	110.81 (6)	N1—C12—N3	109.94 (7)
C12—N1—N2	107.84 (6)	N1—C12—C11	127.14 (7)
C13—N2—N1	106.48 (6)	N3—C12—C11	122.76 (7)
C12—N3—C13	105.17 (6)	N2—C13—N3	110.54 (7)
C12—N3—N4	124.47 (6)	N2—C13—S1	129.92 (6)
C13—N3—N4	128.81 (6)	N3—C13—S1	119.53 (6)
C15—N4—N3	114.39 (6)	C15—C14—S1	110.06 (5)
N6—N5—O2	105.36 (6)	C15—C14—H14A	107.6 (8)
N5—N6—C16	114.66 (6)	S1—C14—H14A	107.9 (8)
N5—N6—C18	115.62 (6)	C15—C14—H14B	114.1 (8)
C16—N6—C18	129.71 (6)	S1—C14—H14B	105.4 (8)
C2—C1—C6	119.52 (8)	H14A—C14—H14B	111.6 (11)
C2—C1—C10	122.14 (8)	N4—C15—C16	118.51 (6)
C6—C1—C10	118.34 (8)	N4—C15—C14	123.04 (7)
C3—C2—C1	120.31 (9)	C16—C15—C14	118.43 (6)
C3—C2—H2A	121.0 (10)	N6—C16—C17	105.06 (6)
C1—C2—H2A	118.6 (10)	N6—C16—C15	126.71 (6)
C2—C3—C4	120.42 (11)	C17—C16—C15	127.69 (6)
C2—C3—H3A	121.6 (11)	O3—C17—O2	120.35 (7)
C4—C3—H3A	117.9 (11)	O3—C17—C16	135.53 (7)
C5—C4—C3	120.13 (10)	O2—C17—C16	104.10 (6)
C5—C4—H4A	120.8 (12)	C19—C18—C23	122.88 (7)
C3—C4—H4A	119.1 (12)	C19—C18—N6	118.68 (7)
C4—C5—C6	121.22 (9)	C23—C18—N6	118.38 (7)
C4—C5—H5A	121.1 (10)	C18—C19—C20	117.98 (7)
C6—C5—H5A	117.6 (10)	C18—C19—H19A	118.5 (8)
C7—C6—C5	121.86 (9)	C20—C19—H19A	123.5 (8)
C7—C6—C1	119.75 (8)	C19—C20—C21	121.14 (8)
C5—C6—C1	118.39 (9)	C19—C20—H20A	117.8 (9)
C8—C7—C6	120.17 (8)	C21—C20—H20A	121.0 (9)
C8—C7—H7A	121.4 (9)	C22—C21—C20	118.96 (8)
C6—C7—H7A	118.4 (9)	C22—C21—C24	120.57 (9)
C7—C8—C9	121.15 (9)	C20—C21—C24	120.44 (9)
C7—C8—H8A	119.6 (9)	C23—C22—C21	121.09 (8)
C9—C8—H8A	119.2 (9)	C23—C22—H22A	118.3 (8)
C10—C9—C8	119.73 (8)	C21—C22—H22A	120.6 (8)
C10—C9—H9A	122.1 (8)	C18—C23—C22	117.92 (8)
C8—C9—H9A	118.1 (8)	C18—C23—H23A	120.7 (8)
O1—C10—C9	124.35 (7)	C22—C23—H23A	121.4 (8)
O1—C10—C1	114.90 (7)	C21—C24—H24A	108.9 (12)
C9—C10—C1	120.74 (7)	C21—C24—H24B	109.1 (11)
O1—C11—C12	109.19 (6)	H24A—C24—H24B	111.9 (16)
O1—C11—H11A	109.2 (8)	C21—C24—H24C	114.4 (10)
C12—C11—H11A	110.5 (8)	H24A—C24—H24C	107.1 (17)
O1—C11—H11B	112.0 (8)	H24B—C24—H24C	105.4 (16)
			
C12—N1—N2—C13	1.48 (8)	C12—N3—C13—N2	0.23 (8)
C12—N3—N4—C15	165.86 (7)	N4—N3—C13—N2	−165.83 (7)
C13—N3—N4—C15	−30.53 (10)	C12—N3—C13—S1	179.55 (5)
C17—O2—N5—N6	−0.43 (8)	N4—N3—C13—S1	13.50 (10)
O2—N5—N6—C16	−0.38 (9)	C14—S1—C13—N2	−153.37 (8)
O2—N5—N6—C18	178.66 (6)	C14—S1—C13—N3	27.46 (6)
C6—C1—C2—C3	−0.97 (16)	C13—S1—C14—C15	−53.80 (6)
C10—C1—C2—C3	178.48 (11)	N3—N4—C15—C16	171.38 (6)
C1—C2—C3—C4	0.6 (2)	N3—N4—C15—C14	−6.97 (10)
C2—C3—C4—C5	0.0 (2)	S1—C14—C15—N4	51.78 (9)
C3—C4—C5—C6	−0.2 (2)	S1—C14—C15—C16	−126.57 (6)
C4—C5—C6—C7	179.19 (13)	N5—N6—C16—C17	1.00 (9)
C4—C5—C6—C1	−0.20 (18)	C18—N6—C16—C17	−177.88 (7)
C2—C1—C6—C7	−178.65 (9)	N5—N6—C16—C15	−171.03 (7)
C10—C1—C6—C7	1.88 (13)	C18—N6—C16—C15	10.09 (12)
C2—C1—C6—C5	0.75 (14)	N4—C15—C16—N6	−15.34 (11)
C10—C1—C6—C5	−178.72 (9)	C14—C15—C16—N6	163.09 (7)
C5—C6—C7—C8	177.25 (11)	N4—C15—C16—C17	174.40 (7)
C1—C6—C7—C8	−3.36 (15)	C14—C15—C16—C17	−7.18 (11)
C6—C7—C8—C9	1.64 (16)	N5—O2—C17—O3	−177.66 (7)
C7—C8—C9—C10	1.61 (15)	N5—O2—C17—C16	1.00 (8)
C11—O1—C10—C9	5.75 (11)	N6—C16—C17—O3	177.21 (9)
C11—O1—C10—C1	−174.30 (7)	C15—C16—C17—O3	−10.86 (15)
C8—C9—C10—O1	176.86 (8)	N6—C16—C17—O2	−1.15 (8)
C8—C9—C10—C1	−3.09 (13)	C15—C16—C17—O2	170.78 (7)
C2—C1—C10—O1	1.93 (12)	N5—N6—C18—C19	−108.58 (8)
C6—C1—C10—O1	−178.61 (7)	C16—N6—C18—C19	70.29 (11)
C2—C1—C10—C9	−178.12 (9)	N5—N6—C18—C23	68.82 (9)
C6—C1—C10—C9	1.34 (12)	C16—N6—C18—C23	−112.31 (9)
C10—O1—C11—C12	−179.64 (6)	C23—C18—C19—C20	−0.86 (12)
N2—N1—C12—N3	−1.37 (9)	N6—C18—C19—C20	176.42 (7)
N2—N1—C12—C11	−176.75 (7)	C18—C19—C20—C21	−0.37 (13)
C13—N3—C12—N1	0.74 (8)	C19—C20—C21—C22	1.50 (13)
N4—N3—C12—N1	167.57 (7)	C19—C20—C21—C24	−176.65 (9)
C13—N3—C12—C11	176.36 (7)	C20—C21—C22—C23	−1.45 (14)
N4—N3—C12—C11	−16.80 (11)	C24—C21—C22—C23	176.69 (9)
O1—C11—C12—N1	−76.16 (10)	C19—C18—C23—C22	0.90 (12)
O1—C11—C12—N3	109.01 (8)	N6—C18—C23—C22	−176.38 (7)
N1—N2—C13—N3	−1.03 (8)	C21—C22—C23—C18	0.29 (13)
N1—N2—C13—S1	179.73 (6)		

### Hydrogen-bond geometry (Å, °)

**Table:**

Cg1 and Cg2 are the centroids of the C18–C23 and C1/C6–C10 benzene rings, respectively.

**Table:**

*D*—H···*A*	*D*—H	H···*A*	*D*···*A*	*D*—H···*A*
C14—H14B···O3	0.973 (13)	2.401 (14)	3.0928 (10)	127.7 (10)
C14—H14B···O3^i^	0.973 (13)	2.396 (13)	3.1612 (10)	135.2 (11)
C8—H8A···Cg1^ii^	0.980 (17)	2.603 (17)	3.3928 (11)	138.0 (13)
C24—H24C···Cg2	0.993 (19)	2.95 (2)	3.7066 (14)	134.3 (17)

Symmetry codes: (i) −*x*, −*y*+2, −*z*+1; (ii) *x*, −*y*+3/2, *z*−3/2.
